# Astragaloside IV ameliorates experimental autoimmune myasthenia gravis by regulating CD4 + T cells and altering gut microbiota

**DOI:** 10.1186/s13020-023-00798-6

**Published:** 2023-08-04

**Authors:** Senhui Weng, Linwen Huang, Bingxing Cai, Long He, Shuting Wen, Jinghao Li, Zhuotai Zhong, Haiyan Zhang, Chongyang Huang, Yunying Yang, Qilong Jiang, Fengbin Liu

**Affiliations:** 1 Department of Spleen and Stomach Diseases, Guangdong Provincial Hospital of Chinese Medicine, No. 111 Dade Road, Yuexiu District, Guangzhou, 510120 China; 2 Guangzhou University of Chinese Medicine, No.12 Airport Road, Baiyun District, Guangzhou, 510422 China; 3 Lingnan Medical Research Center of Guangzhou University of Chinese Medicine, No.12 Airport Road, Baiyun District, Guangzhou, 510422 China; 4 Department of Traditional Chinese Medicine of the Sixth Affiliated Hospital, School of Medicine, South China University of Technology, Foshan, 528000 China; 5 Department of Spleen and Stomach Diseases, First Affiliated Hospital of Guangzhou University of Chinese Medicine, No.16 Airport Road, Baiyun District, Guangzhou, 510422 China; 6 Baiyun Hospital of the First Affiliated Hospital of Guangzhou University of Chinese Medicine, No. 2, Helongqi Road, Renhe Town, Baiyun District, Guangzhou, 510000 China

**Keywords:** Astragaloside IV, Gut microbiota, Experimental autoimmune myasthenia gravis, T cell, Fecal microbial transplantation

## Abstract

**Background:**

Myasthenia gravis (MG) is an antibody-mediated autoimmune disease and its pathogenesis is closely related to CD4 + T cells. In recent years, gut microbiota is considered to play an important role in the pathogenesis of MG. Astragaloside IV (AS-IV) is one of the main active components extracted from Astragalus membranaceus and has immunomodulatory effects. To study the immunomodulatory effect of AS-IV and the changes of gut microbiota on experimental autoimmune myasthenia gravis (EAMG) mice, we explore the possible mechanism of AS-IV in improving MG.

**Methods:**

In this study, network pharmacology was utilized to screen the crucial targets of AS-IV in the treatment of MG. Subsequently, a Kyoto Encyclopedia of Genes and Genomes (KEGG) enrichment analysis was performed to identify potential pathways through which AS-IV acts against MG. Furthermore, experimental investigations were conducted to validate the underlying mechanism of AS-IV in MG treatment. Before modeling, 5 mice were randomly selected as the control group (CFA group), and the other 10 were induced to EAMG model. These mice were randomly divided into EAMG group and EAMG + AS-IV group, n = 5/group. In EAMG + AS-IV group, AS-IV was administered by gavage. CFA and EAMG groups were given the same volume of PBS. Body weight, grip strength and clinical symptoms were assessed and recorded weekly. At the last administration, the feces were collected for 16S RNA microbiota analysis. The levels of Treg, Th1 and Th17 cells in spleen and Th1 and Th17 cells in thymus were detected by flow cytometry. The levels of IFN-γ, IL-17 and TGF-β in serum were measured by ELISA. Furthermore, fecal microbial transplantation (FMT) experiments were performed for exploring the influence of changed intestinal flora on EAMG. After EAMG model was induced, the mice were treated with antibiotics daily for 4 weeks to germ-free. Then germ-free EAMG mice were randomly divided into two groups: FMT EAMG group, FMT AS-IV group, n = 3/group. Fecal extractions from EAMG and EAMG + AS-IV groups as gathered above were used to administered daily to the respective groups for 4 weeks. Body weight, grip strength and clinical symptoms were assessed and recorded weekly. The levels of Treg, Th1 and Th17 cells in spleen and Th1 and Th17 cells in thymus were detected at the last administration. The levels of IFN-γ, IL-17 and TGF-β in serum were measured by ELISA.

**Results:**

The network pharmacology and KEGG pathway analysis revealed that AS-IV regulates T cell pathways, including T cell receptor signaling pathway and Th17 cell differentiation, suggesting its potential in improving MG. Further experimental verification demonstrated that AS-IV administration improved muscle strength and body weight, reduced the level of Th1 and Th17 cells, enhanced the level of Treg cells, and resulted in alterations of the gut microbiota, including changes in beta diversity, the Firmicutes/Bacteroidetes (F/B) ratio, and the abundance of Clostridia in EAMG mice. We further conducted FMT tests and demonstrated that the EAMG Abx-treated mice which were transplanted the feces of mice treated with AS-IV significantly alleviated myasthenia symptoms, reduced Th1 and Th17 cells levels, and increased Treg cell levels.

**Conclusion:**

This study speculated that AS-IV ameliorates EAMG by regulating CD4 + T cells and altering the structure and species of gut microbiota of EAMG.

## Introduction

Myasthenia gravis (MG) is an acquired autoimmune disease mediated by autoantibodies targeting neuromuscular junctions, resulting in muscle weakness and fatigue. It is a rare disease with 0.3 to 2.8 cases per million person-years [1, 2] and a prevalence of 77.7 cases per million people [3]. Approximately 85% of MG patients have circulating autoantibodies that bind to the acetylcholine receptor (AChR) [4]. Although antibodies are directly produced by B cells, the pathogenesis of MG is closely correlated with CD4 + T cells and their cytokines which promote antigen-experienced B cells to produce pathogenic antibodies. Native CD4 + T cells can be functionally divided into four subsets: T helper cells 1 (Th1), Th2, Th17, and regulatory T (Treg) cells. Voluminous data indicate that Th1, Th17, and Treg cells are involved in the development of MG, with complex correlations of interactions among the cells and their cytokines [5].

Studies have demonstrated that MG patients have higher levels of Th1 and Th17 cells and lower levels of Treg cells than healthy individuals in the peripheral blood [6–8]. Th1 cells which produce IFN-γ mainly activate antigen-presenting cells and facilitate cellular immune responses. Th17 cells break the balance of cytokines produced by Th1 and Th2 cells and consequently affect the production of antibodies in MG patients [9]. IL-17A, which is almost produced by Th17 cells, is increased in the peripheral blood in MG patients and correlated with the severity of MG [10]. Treg cells, which express the transcription factor forkhead box protein 3 (Foxp3), play a pivotal role in maintaining peripheral tolerance to self-antigens and regulating Th and B cell immune responses. The level of Treg cells in MG patients is decreased and defective, and the proportion of Treg cells is relevant to the Quantitative Myasthenia Gravis (QMG) score, which is used to evaluate MG severity [8].

Altogether, the imbalance of these T cell subsets (Th1/ Th17/ Treg) and their cytokines may contribute to the pathogenesis and exacerbation of MG and correlate with clinical parameters in MG patients [11–13]. In addition, some studies have shown that gut microbiota plays a role in the pathogenesis and development of MG. A tremendous amount of resident microbiota parasitizes the gut, constituting complex and dynamic ecosystems as a massive source of antigenic variation, for which the immune system must control its response. The gut microbiota plays an essential role in the immune cells. Studies have shown that CD4 + T cells participate in complex and dynamic cross-communication with the gut microbiota to coordinate immune responses [14]. Recent studies have shown that gut microbiota dysbiosis in MG patients was characterized by a reduction in alpha diversity, an increased count of certain bacteria such as Clostridium, and a decreased level of short-chain fatty acids (SCFAs) in the intestine, which aggravates the severity of the disease [15].

Astragaloside IV (AS-IV, 3-O-beta-D-xylopyranosyl-6-O-beta-D-glucopyranosyl-cycloastragenol) is a saponin isolated from Astragalus membranaceus (AM, *Huangqi*). It has a wide range of pharmacological activities, including antioxidant, anti-inflammatory, cardioprotective, antiviral, antibacterial, antifibrotic, and immunoregulatory effects [16]. A study showed that AS-IV could regulate Th1/Th2 cytokines and raise the level of Treg cells, attenuating allergic inflammation and thereby ameliorating asthma [17]. Moreover, AS-IV significantly relieved experimental autoimmune encephalomyelitis (EAE), downregulated Th1 and Th17 cells, and upregulated Treg cell levels [18]. The pathogenesis of MG is relevant to CD4 + T cells, thus in the current study, we expected to investigate whether AS-IV ameliorates experimental autoimmune myasthenia gravis (EAMG) symptoms and affects EAMG by modulating the subsets of CD4 + T cells. In addition, we investigated how AS-IV treatment modulates gut microbiota during EAMG using 16S rRNA. Lastly, we performed fecal microbial transplantation (FMT) experiments to reveal that alterations in the gut microbiota after treatment with AS-IV can regulate T cells and improve the clinical symptoms of EAMG. Thus, we hypothesized that AS-IV might affect the expression of T cells and influence the composition of intestinal flora, thus affecting the progression of EAMG disease, which may contribute to the clinical application of AS-IV in the prevention and treatment of MG.

## Materials and methods

### Collection of potential targets for AS-IV

First, we searched the PubChem database (https://pubchem.ncbi.nlm.nih.gov/) for the molecular structure of AS-IV. Then, potential targets of AS-IV were predicted by the Pharm Mapper Server (http://lilab.ecust.edu.cn/pharmmapper/) and the SwissTargetPrediction database (http://www.swisstargetprediction.ch/). Finally, only targets with a fit score greater than 0.6000 were reserved, and the different ID types of the targets were converted to UniProt IDs.

### Collection of potential targets for MG

Potential targets for MG were collected from the GeneCards database (http://www.genecards.org/) and the OMIM database (http://www.omim.org/).

### Network construction and analysis

The AS-IV and MG-related gene sets were obtained by intersecting the AS-IV target gene set and the MG-related gene set, which were then imported into Cytoscape (version 3.8.2) for visualization.

### KEGG enrichment analysis

The Kyoto Encyclopedia of Genes and Genomes (KEGG) pathway analysis revealed the underlying mechanisms through crucial signaling pathways. The R 3.6.2 software and packages of Bioconductor, which included org. Hs.eg.db, clusterProfile, pathview, and ggplot2 were used for the KEGG pathway analysis (p < 0.05) of the AS-IV and MG-related gene set above and visualizing the results.

### Experimental animals

Female C57BL/6 mice, 8–10 weeks of age, were purchased from Guangdong Medical Laboratory Animal Center with license No. SCNK (Guangdong) 2018–0002. All mice were maintained under specific pathogen-free conditions (22.2 °C with 35–55% relative humidity on a 12 h light/dark cycle) with free access to food and water. The mice were placed in a specific pathogen-free (SPF) facility. All in vivo experiments were performed in accordance with protocols approved by the Institutional Animal Ethics Committee of the First Affiliated Hospital of Guangzhou University of Chinese Medicine (Ethics No. TCMF1-2021012).

### Experimental autoimmune myasthenia gravis model

The α97-116 peptide of the rat AChR α-subunit (R97-116, DGDF AIVK FTKV LLDY TGHI, synthesized by GL Biochem Ltd., Shanghai, China) was used to induce EAMG. Briefly, mice in the experimental groups were anesthetized and immunized subcutaneously at the base of the tail with 50 μg R97-116 peptide in 200 μL complete Freund’s adjuvant (CFA) supplemented with 1 mg of Mycobacterium tuberculosis strain H37RA on day 0 and boosted on day 30 and day 45 with the same peptide in incomplete Freund’s adjuvant (IFA) [19]. The control mice, assigned as the CFA group, were given the same emulsion except that PBS was used instead of the peptide. The severity of EAMG, indicated by the extent of muscle weakness, was graded 0–4 based on the presence of tremor, hunchback posture, muscle strength, and fatigability [20]. The mice were randomly divided into three groups: (1) EAMG + AS-IV group, AS-IV at 20 mg/kg dose dissolved in 200 μL PBS solution per day. (2) EAMG group, 200 μL PBS solution per day. (3) CFA group, 200 μL PBS solution per day. Clinical assessment, weight and myasthenic scoring of the mice in each group were recorded each week during the experiment.

### Preparation of mononuclear cells (MNCs) from spleen and thymus and flow cytometry analysis

We grinded tissues through a cell strainer (Becton Dickenson, Franklin Lakes, NJ, USA) to prepare MNCs from spleens and thymuses of mice. The MNCs were then isolated by gradient centrifugation, and washed for three times before being resuspended in RPMI 1640 (HyClone, Beijing, China). Then MNCs were stained with anti-CD4 FITC and anti-CD25 PE, after fixation and permeabilization, cells were labeled with anti-IFN-γ PE, anti-IL-17 PE, and anti-FOXP3 APC. The percentages of CD4 + IFN-γ + (Th1), CD4 + IL-17 + (Th17), and CD4 + Foxp3 + (Treg) cells were detected by flow cytometry. The data were analyzed by FlowJo V.10 software (Treestar Inc., Ashland, OR, USA). All the reagents required above were purchased from Tonbo Biosciences (San Diego, CA, USA).

### Detection of cytokine and anti-R97-116 IgG

The concentrations of cytokines TGF-β (DG30601M-96), IL-17 (DG30110M-96), and IFN-γ (DG30748M-96) were measured using ELISA kits according to the manufacturer’s instructions (Dogesce, Beijing, China). And the titers of anti-R97-116 IgG was determined as described previously [19, 21]. We coated AChR R97-116 (5 μg/ml in 100 μL) on the 96-well flat-bottomed plates at 4℃ overnight and washed the next day and blocked. Next, serum (1:500) were incubated and then washed for 5 times. Next, we added HRP-conjugated rabbit anti-rat IgG (1:2000) and incubated at 37℃ for 1 h. Finally, the reaction developed in the dark at indoor temperature. The absorbance was measured at 450 nm using a microplate reader (Thermo Scientific, Waltham, MA, USA). The analyses were performed in duplicate, and the cytokine results were expressed as the mean cytokine concentration (pg/ml) ± SD and the anti-R97-116 IgG titer results expressed as OD value ± SD 16S rRNA Sequencing and Bioinformatic Analysis.

### 16S rRNA sequencing and bioinformatic analysis

Cecal flushes were collected from CFA, EAMG, and EAMG + AS-IV mice under antiseptic conditions, and collected samples were kept frozen at -80℃ until DNA extraction. Genomic DNA was extracted from stool samples and quantified. Then all the quality genomic DNA samples were used to construct libraries for PCR-based amplification. The V4 region of the 16S rRNA gene was amplified using the 515F-806R primer (515F: GTGCCAGCMGCCGCGGTAA; 806R: GGACTACHVGGGTWTCTAAT). A PCR thermocycler (GeneAmp 9700; Applied Biosystems, France) was used to perform PCR-based amplification. The qualified library was used for sequencing in Illumina HiSeq 2500 platform (Illumina, San Diego, CA, USA). QIIME (version 1.9.1) was used to demultiplex and quality-filter the raw FASTQ files for 16S analysis. Further, operational taxonomic units (OTUs) were generated and clustered with a 97% similarity by using UPARSE and the OTU unique representative sequences were obtained. After aligning with the gold database (v20110519), chimaeric sequences were screened using UCHIME (version 4.2.40), and the OTU abundances of each sample was quantified using Usearch_global (version 7.1). Each representative OTU was taxonomically classified using Ribosomal Database Project Classifier (version 2.2) trained on the Greengenes database (v201305). OTUs were then divided into virous hierarchical levels, and the taxonomic relative abundance profiles were summarized. Alpha diversity described the within sample diversity. Beta diversity described the bacterial community dissimilarity among different sample communities.

### Gut microbiota depletion and fecal microbial transplantation (FMT)

To perform FMT tests, EAMG were induced as described above. Fresh feces from EAMG, and EAMG + AS-IV groups were collected aseptically using an anaerobic chamber and suspended in sterile PBS containing 30% glycerol for storage at -80 °C until used. For preparing EAMG mice for FMTs, mice were administered antibiotics (ampicillin 1 g/L, neomycin 1 g/L, vancomycin 0.5 g/L, and metronidazole 1 g/L, referred to as “Abx”) by oral gavage daily for four weeks. EAMG Abx-treated mice were randomized into 2 groups: FMT EAMG, FMT AS-IV, n = 3/group. FMTs (50 mg) were administered daily by intragastric administration in EAMG Abx-treated mice for four weeks.

### Statistical analysis

Statistical analyses were carried out using GraphPad Prism 9.1.2 (GraphPad Inc., La Jolla, CA, USA). Statistical significance was set at p < 0.05. For continuous variables, unpaired two-tailed t-test or one-way analysis of variance (ANOVA) followed by Tukey’s multiple comparisons tests was carried out for analyses. Non-parametric factorial Wilcoxon rank-sum test or Kruskal–Wallis test followed by Dunn’s multiple comparisons tests to compare two or three groups would be used in case that the data was heteroscedasticity or non-normally distributed variables. And p values were stated as follows: *p < 0.05, **p < 0.01, ***p < 0.001; ns, not significant (p > 0.05).

## Results

### AS-IV and MG target collection and KEGG enrichment analysis through network pharmacology

**Fig. 1:**
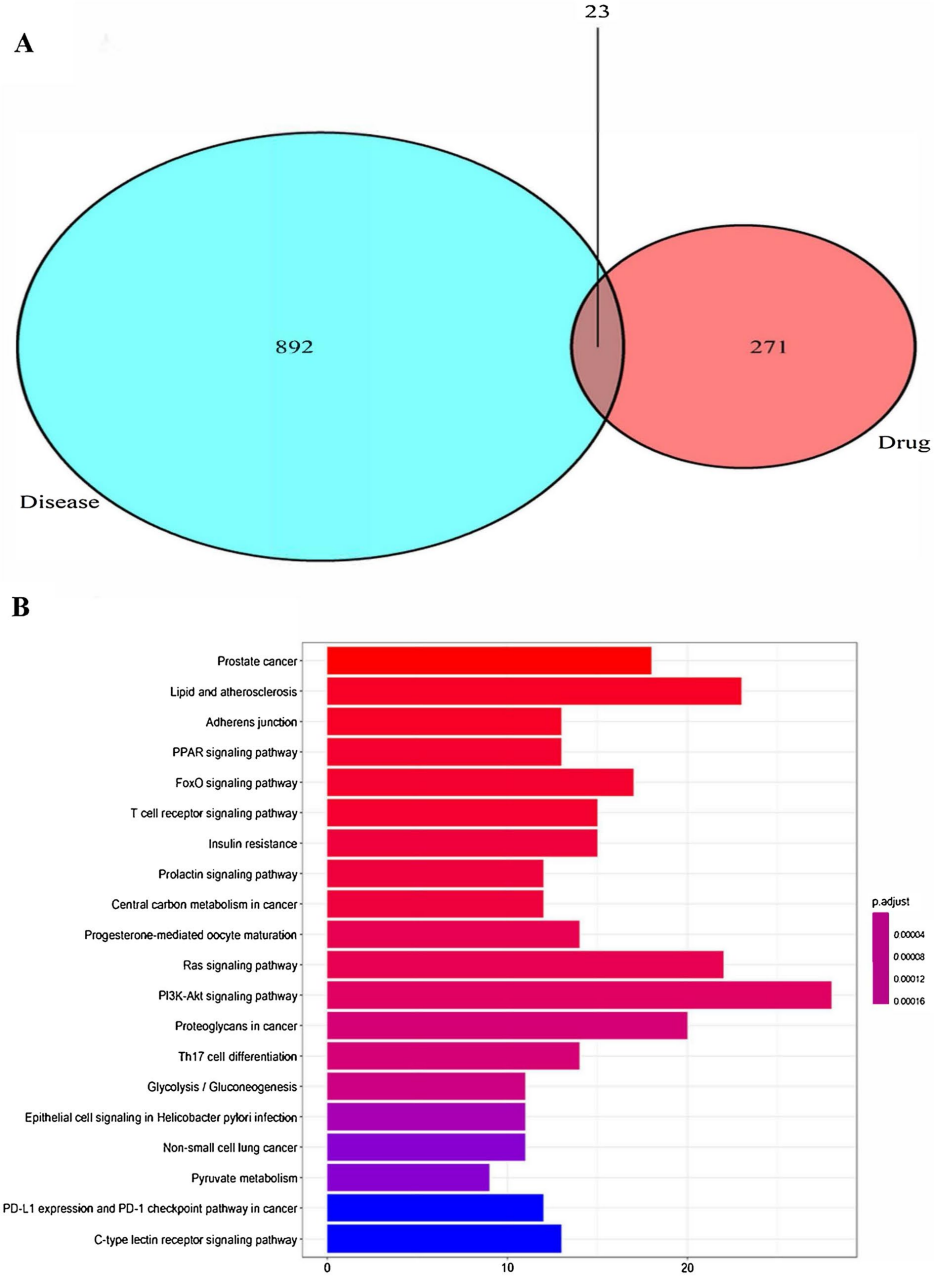
**A** Identification of the drug-target disease-related genes by taking an intersection of AS-IV target genes and MG-related genes. **B** KEGG pathway enrichment analysis of the target genes

To explore whether AS-IV affect MG through T cell-related pathways, network pharmacology was performed in this study. The Pharm Mapper databases and Swiss Target Prediction database were used to screen 271 targets corresponding to AS-IV. In addition, a total of 892 MG-associated targets were identified based on the OMIM and Genecard databases. Further, 23 intersection genes were obtained from AS-IV and MG target genes by drawing a Venn diagram (Fig. 1A), which are possible targets for AS-IV in the treatment of MG. String database was performed to obtain the protein and protein interactions of those 23 intersection genes. The result was then analyzed by using Cytoscape 3.8.2 software and the PPI network was constructed.

To further clarify the mechanism underlying the effects of AS-IV on MG, we conducted the KEGG pathway enrichment analysis on these 23 protein targets. A bubble chart was constructed to display the top 20 KEGG pathways screened by p-values (Fig. 1B). Based on this analysis, we found T cell-related pathways, such as the T cell receptor signaling pathway and Th17 cell differentiation. Therefore, AS-IV is likely to treat MG by regulating T cells.

### AS-IV ameliorated EAMG symptoms and reduced anti-R97-116 IgG titer in EAMG

**Fig. 2:**
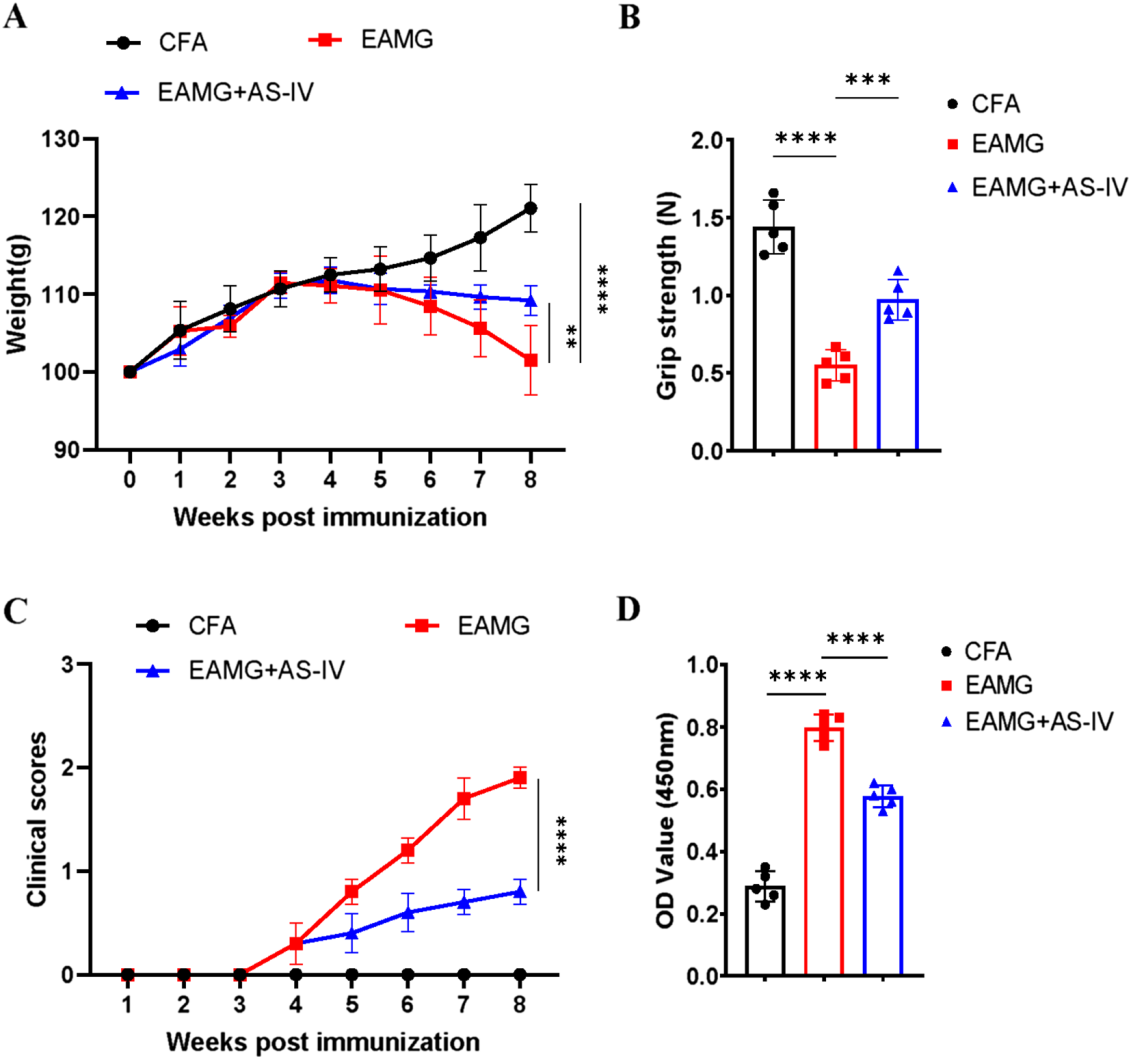
AS-IV improved EAMG. Intraperitoneal administration of AS-IV (20 mg/kg) was given to EAMG once daily beginning on post-immunization. n = 5/group. Body weight (**A**), grip strength (**B**), and clinical scores (**C**) were measured in mice of the CFA, EAMG, and EAMG + AS-IV group. **D** The anti-R97-116 IgG titers in serum from mice in CFA, EAMG, and EAMG + AS-IV group were determined by ELISA. Data are expressed as the mean ± SD, *p < 0.05, **p < 0.01, ***p < 0.001, ****p < 0.0001

Weight loss (Fig. 2A), grip strength decrease (Fig. 2B), and clinical score increase (Fig. 2C) were our outcome observations, which show the severity of EAMG. Lower clinical scores, higher grip strength, and less weight loss were showed significantly in EAMG + AS-IV group. The titers of anti-R97-116 IgG in the serum of mice were detected by ELISA (Fig. 2D). The mice in EAMG + AS-IV group had a significantly lower titers of anti-R97-116 IgG than the EAMG group. All these suggested that AS-IV has a therapeutic effect on EAMG.

### AS-IV down-regulated Th1, Th17, and up-regulated Treg proportions in MNCs from EAMG

**Fig. 3:**
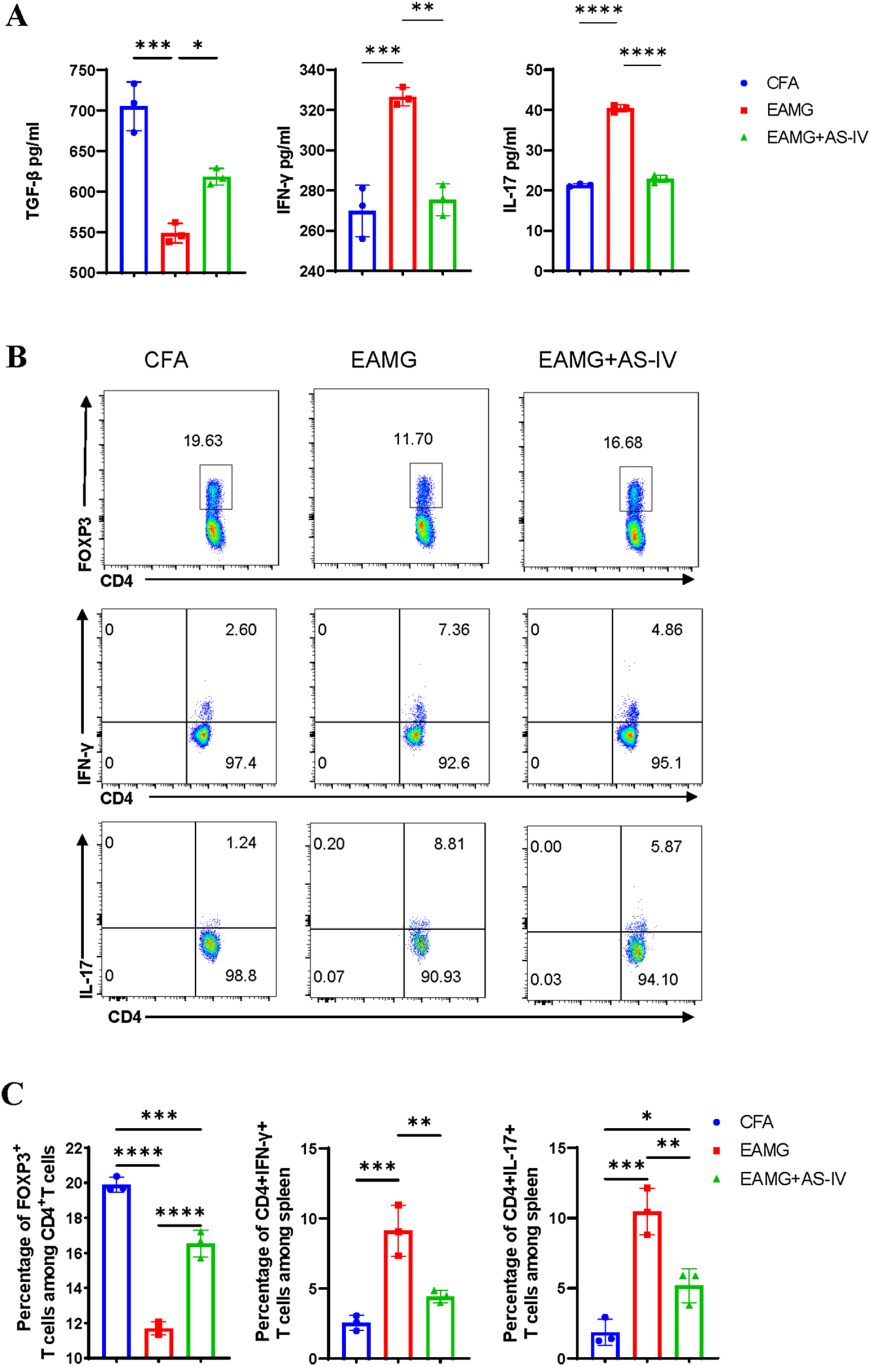

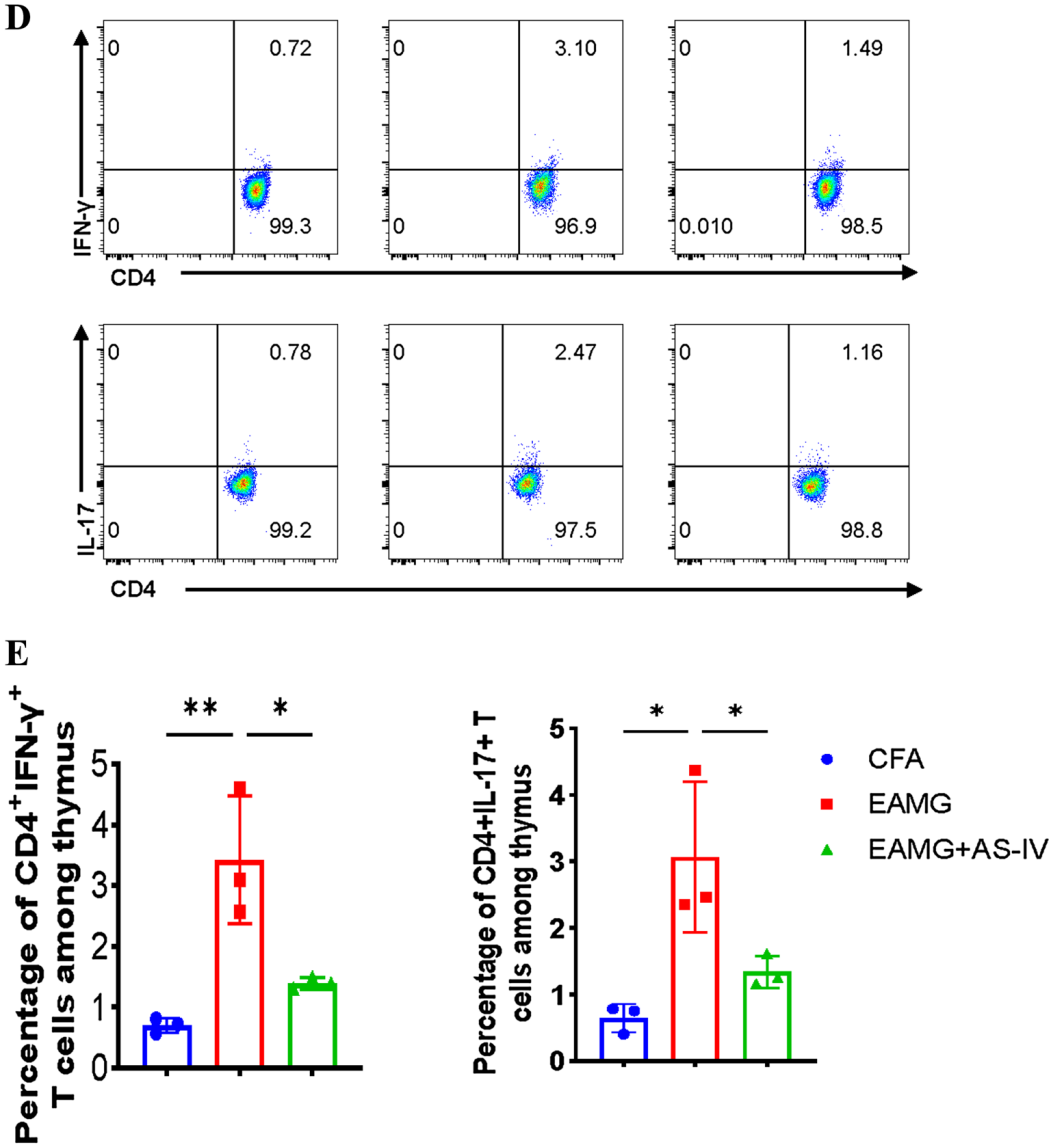
AS-IV altered CD4 + T cell subsets distribution and cytokines secretion in vivo. The serum were obtained from mice of CFA, EAMG, EAMG + AS-IV groups after 8 weeks post immunization, and cytokines levels of TGF-β, IFN-γ, and IL-17 were detected by ELISA (**A**). Mononuclear cells (MNCs) of the spleens and thymus were isolated from mice in three groups after 8 weeks post immunization. CD4 + Foxp3 + (Treg) cells, CD4 + IFN-γ + (Th1) cells, and CD4 + IL-17 + (Th17) cells were detected by flow cytometry (**B**, **D**). The percentages of Treg, Th1 and Th17 cells in MNCs were calculated **C**, **E**, n = 3/group. The data are representative of three independent experiments (*p < 0.05, **p < 0.01, ***p < 0.001, ****p < 0.0001)

We explored the effect of AS-IV on CD4 + T cells in the EAMG. As explained above, AS-IV can improve autoimmune diseases, such as EAE, by regulating T cells. We counted the number of Th1 and Th17 cells in the spleen and thymus and Treg cells in the spleen. The peripheral blood of mice was collected, and the levels of the cytokines (IL-17, IFN-γ, and TGF-β) in serum were analyzed. Consequently, we found that EAMG + AS-IV group had higher levels of serum TGF-β and lower levels of IL-17 and IFN-γ compared with EAMG group (Fig. 3A). In addition, we found that compared with CFA group, Th1 and Th17 cells were increased in the spleen and thymus, and Treg cells in the spleen were decreased in EAMG group, whereas AS-IV mitigated this trend (Fig. 3B–E). This suggests that the Th1 and Th17 cells and their producing cytokines were higher, whereas the Treg cells and their secreting cytokines were lower in EAMG than in CFA; however, AS-IV treatment ameliorated this trend. Therefore, we found that AS-IV ameliorated EAMG by regulating the imbalance of Th1, Th17, and Treg cells in EAMG mice.

### Effect of AS-IV on the alteration of the gut microbiota

**Fig. 4:**
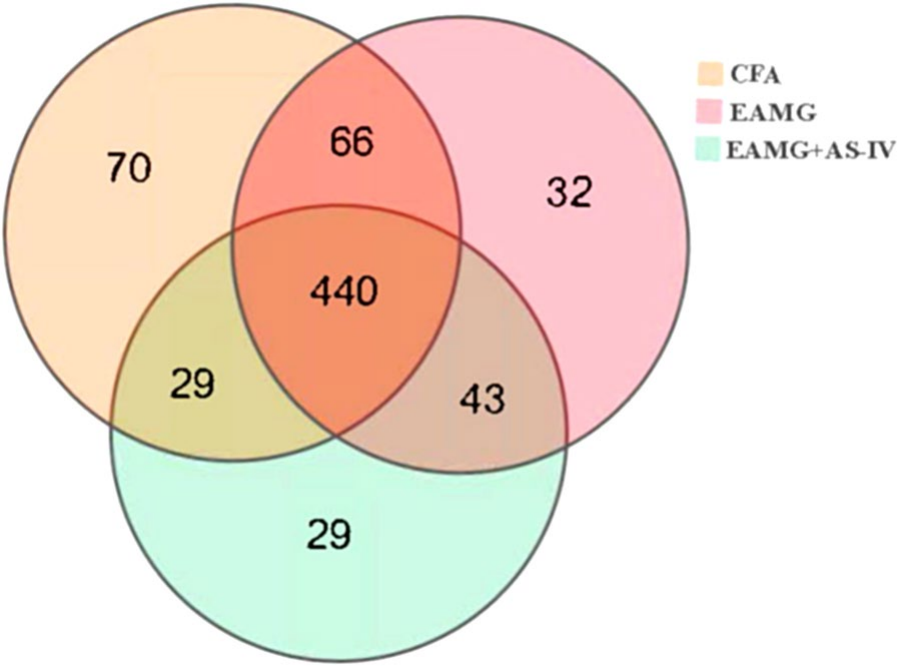
Comparison of the gut microbial composition among the three groups at family and OTU levels. A Venn diagram demonstrated that 440 of 709 OTUs were discovered among the three groups, whereas 70, 32 and 29 OTUs were specific to CFA (yellow circle), EAMG (pink circle), and EAMG + AS-IV (green circle), respectively

Since the gut microbiota may play an important role in MG pathogenesis, we then determined whether AS-IV affects the gut microbiota in the mice model. To estimate the potential role of gut microbiota in AS-IV-mediated attenuation of EAMG, 16S rRNA gene sequencing was performed on gut microbiota from the following groups: CFA, EAMG, and EAMG + AS-IV. The Venn diagram showed that 440 OTUs overlapped among the three groups, and the distinctive OTU numbers in the CFA, EAMG, and EAMG + AS-IV group were 70, 32, and 29, respectively (Fig. 4).

**Fig. 5:**
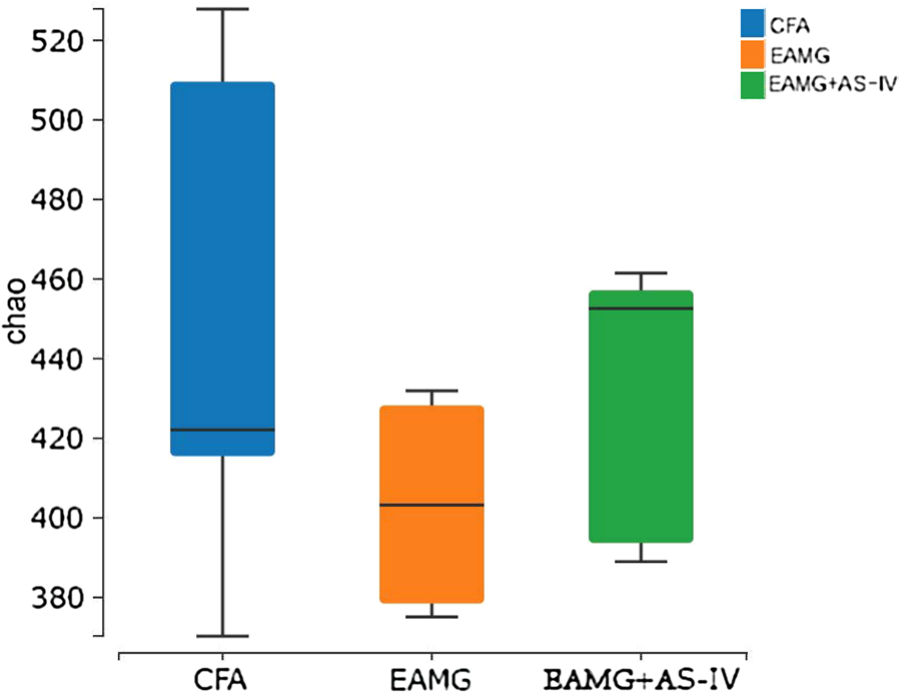
Alpha diversity boxplot (Chao indexes) among CFA, EAMG, and EAMG + AS-IV group

**Fig. 6:**
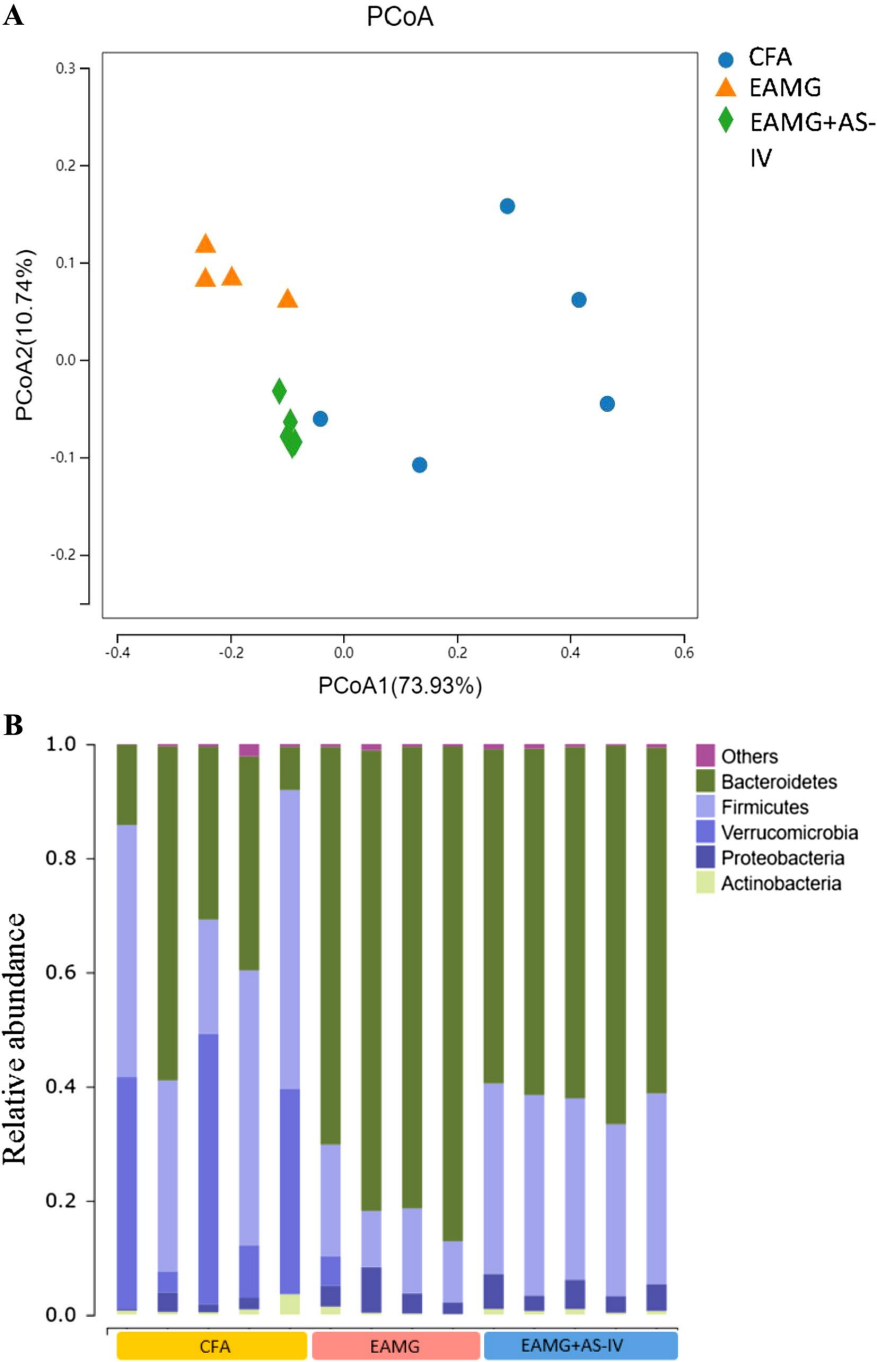
**A** Beta diversity PCoA plots. **B** Analysis of the fecal microbiota composition of mice in the CFA, EAMG and EAMG + AS-IV groups at the phylum level (relative abundance)

**Fig. 7:**
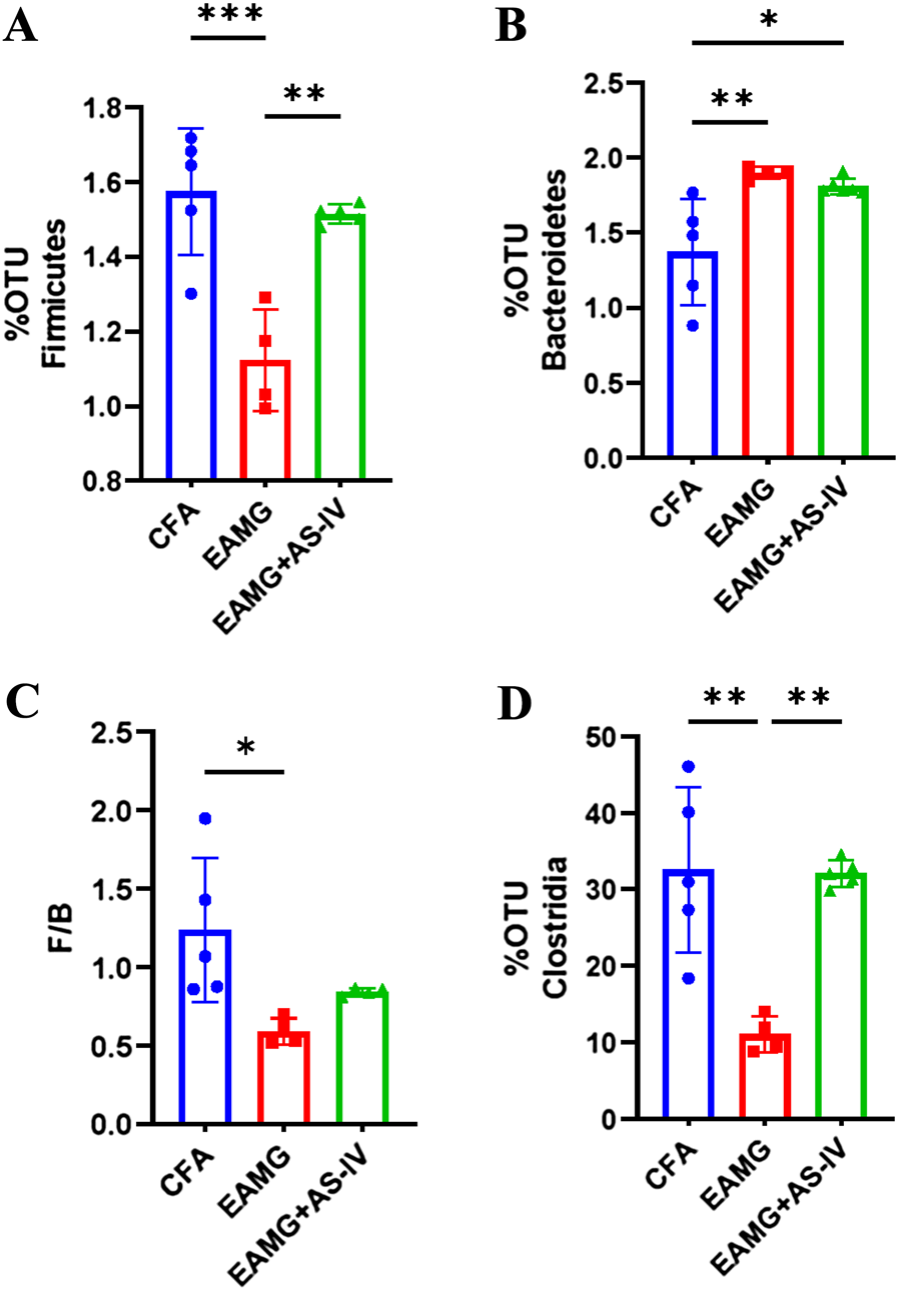
Percent OTUs of Firmicutes **A** and Bacteroidetes **B** and the F/B radio (**C**). **D** Percent OTUs of Clostridia at the class level. Significance was appointed using one-way ANOVA and Tukey’s multiple comparisons test. Bar graph data are expressed as the mean ± SD and statistical significance is indicated as *p < 0.05, **p < 0.01, ***p < 0.001 and ****p < 0.0001

Alpha diversity, as assessed using the Chao1 rarefaction measure, showed no significant difference in abundances of bacteria in the gut among CFA, EAMG, and EAMG + AS-IV group (Fig. 5). Beta diversity, depicted in a principal coordinate analysis (PCoA) plot, showed that the three groups clustered together well within their groups, suggesting a divergence in the gut microbiome population in weighted (Fig. 6A). At the phylum level (Fig. 6B), we found that Firmicutes in the EAMG group were significantly lower than those in the CFA group (p < 0.001), while AS-IV treatment clearly enhanced the abundance of Firmicutes (p < 0.01) (Fig. 7A). In contrast, the abundance of Bacteroidetes in the EAMG and EAMG + AS-IV was significantly greater than that in the CFA group (Fig. 7B). Compared with CFA group, the F/B ratio in EAMG decreased significantly (Fig. 7C). Furthermore, at the class level, the abundance of Clostridia in EAMG was significantly decreased than CFA, whereas AS-IV treatment had risen it (Fig. 7D).

**Fig. 8:**
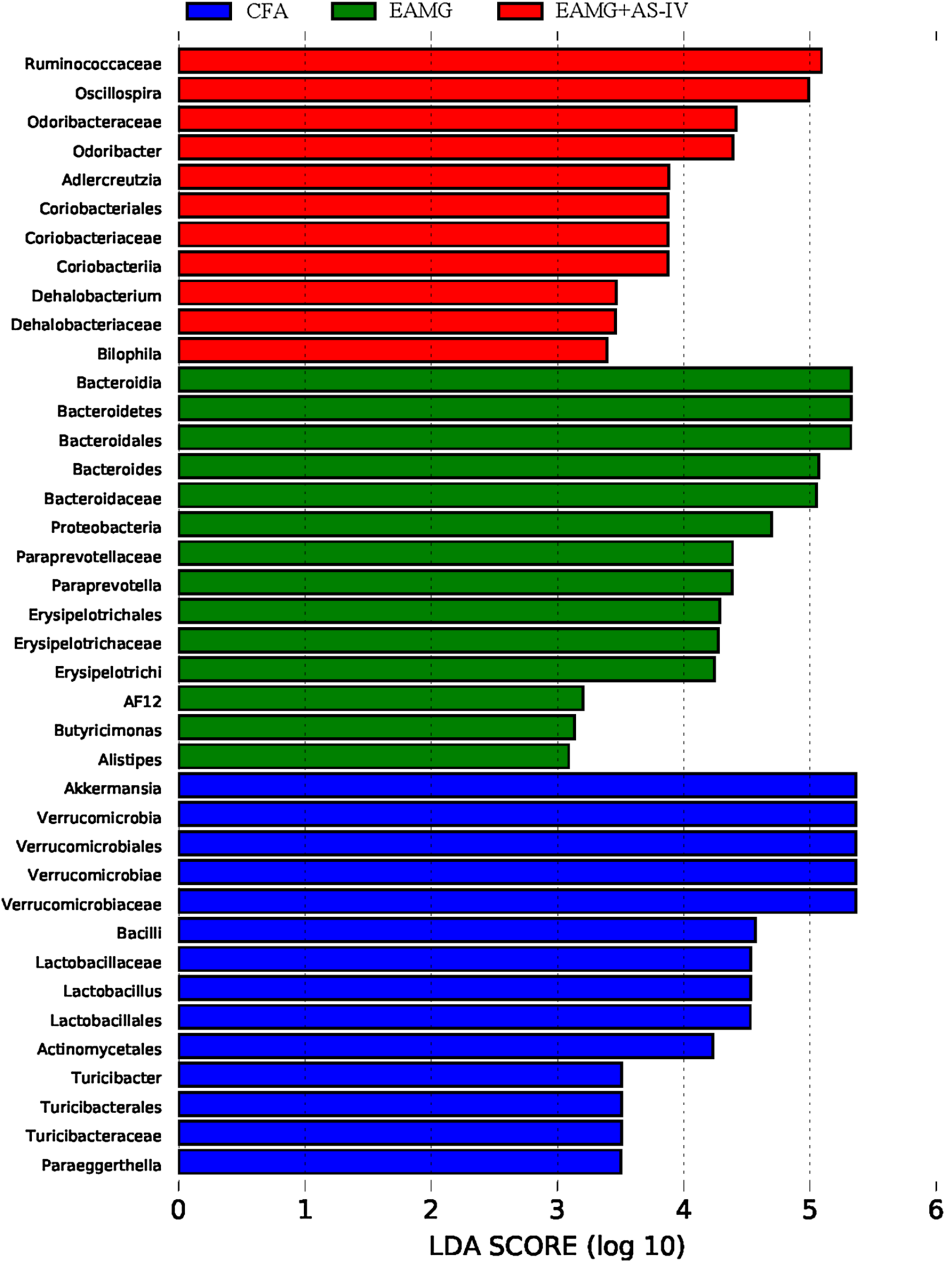
LEfSe of species with significant abundance differences among CFA, EAMG, and EAMG + AS-IV group. The linear discriminant analysis (LDA) threshold for discriminative features was set as 3

In order to ascertain the most significantly altered bacteria between CFA and disease controls and identify any potential microbial biomarkers of disease, linear discriminant analysis effect size (LEfSe) was performed on the OTU output data. LEfSe was performed using the 16S rRNA gene profiling data to identify the phylotypes responsible for the differences among CFA, EAMG, and EAMG + AS-IV. At the genus level, significant increases in Bacteroides and Paraprevotella in the EAMG group compared with the CFA group. In contrast, these bacteria were decreased in the EAMG + AS-IV group compared to the EAMG group. Additionally, Oscillospira showed a slight decrease in the EAMG group compared to the CFA group, but a significant increase in the EAMG + AS-IV group compared to the EAMG group. Furthermore, we observed sharp decreases in Akkermansia and Lactobacillus in both the EAMG and EAMG + AS-IV groups when compared to the CFA group (Fig. 8).

### FMT of AS-IV microbiome improved EAMG symptoms and regulated Th1, Th17, and Treg

**Fig. 9:**
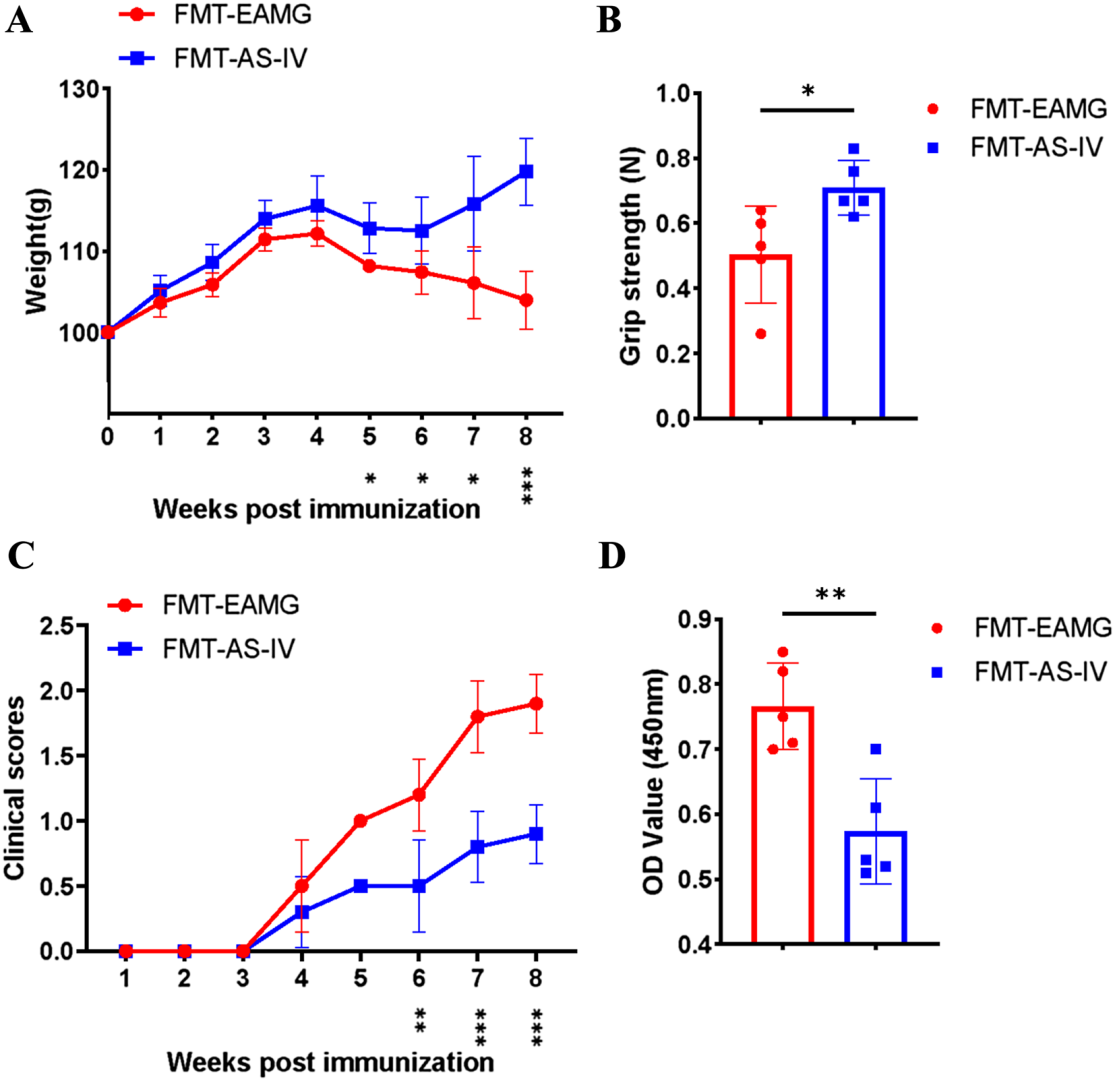
FMT of AS-IV altered microbiome attenuates EAMG severity. Body weight (**A**), grip strength (**B**), clinical scores (**C**), and serum anti-R97-116 IgG titer of mice (**D**) in FMT EAMG group (n = 3) and FMT AS-IV group (n = 3) were compared. Significance was determined using two-way unpaired t-test to evaluate significance at each week. Significant p values (*p < 0.05, **p < 0.01, ***p < 0.001 and ****p < 0.0001) are signaled below corresponding days after EAMG induction

FMT experiments were performed to determine whether the alterations in the gut microbiota by AS-IV treatment were responsible for any positive effects on EAMG onset. For FMTs, EAMG-induced mice were fed fecal material from EAMG control (FMT EAMG) or EAMG treated with AS-IV (FMT AS-IV). In this study, we found that FMT- AS-IV group had more weight (Fig. 9A), greater grip strength (Fig. 9B), lower clinical scores (Fig. 9C), and lower anti-R97-116 IgG titers in the serum than FMT EAMG group (Fig. 9D). These data confirmed that alterations in the microbiome by AS-IV may have a potential but essential role in protection on EAMG onset.

**Fig. 10:**
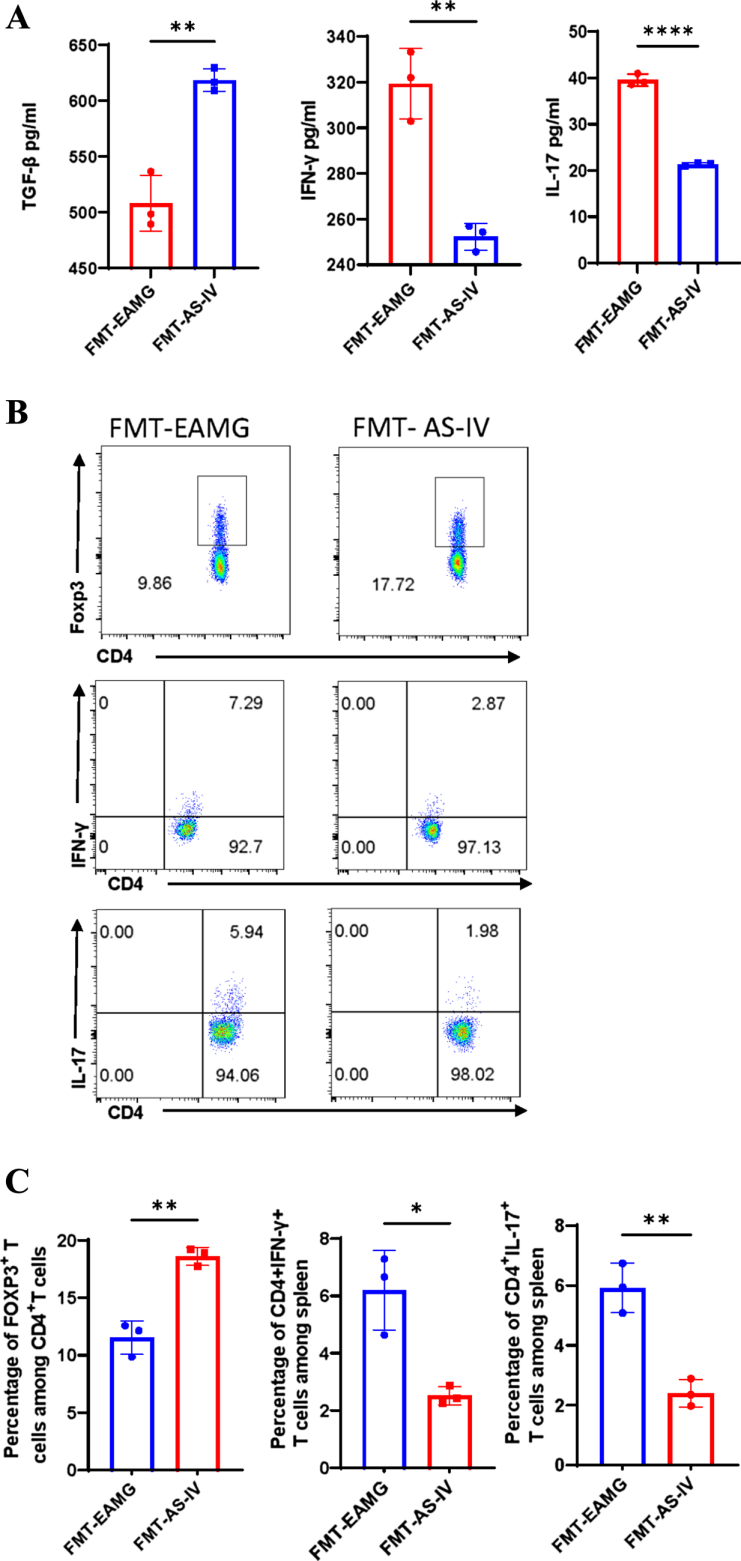

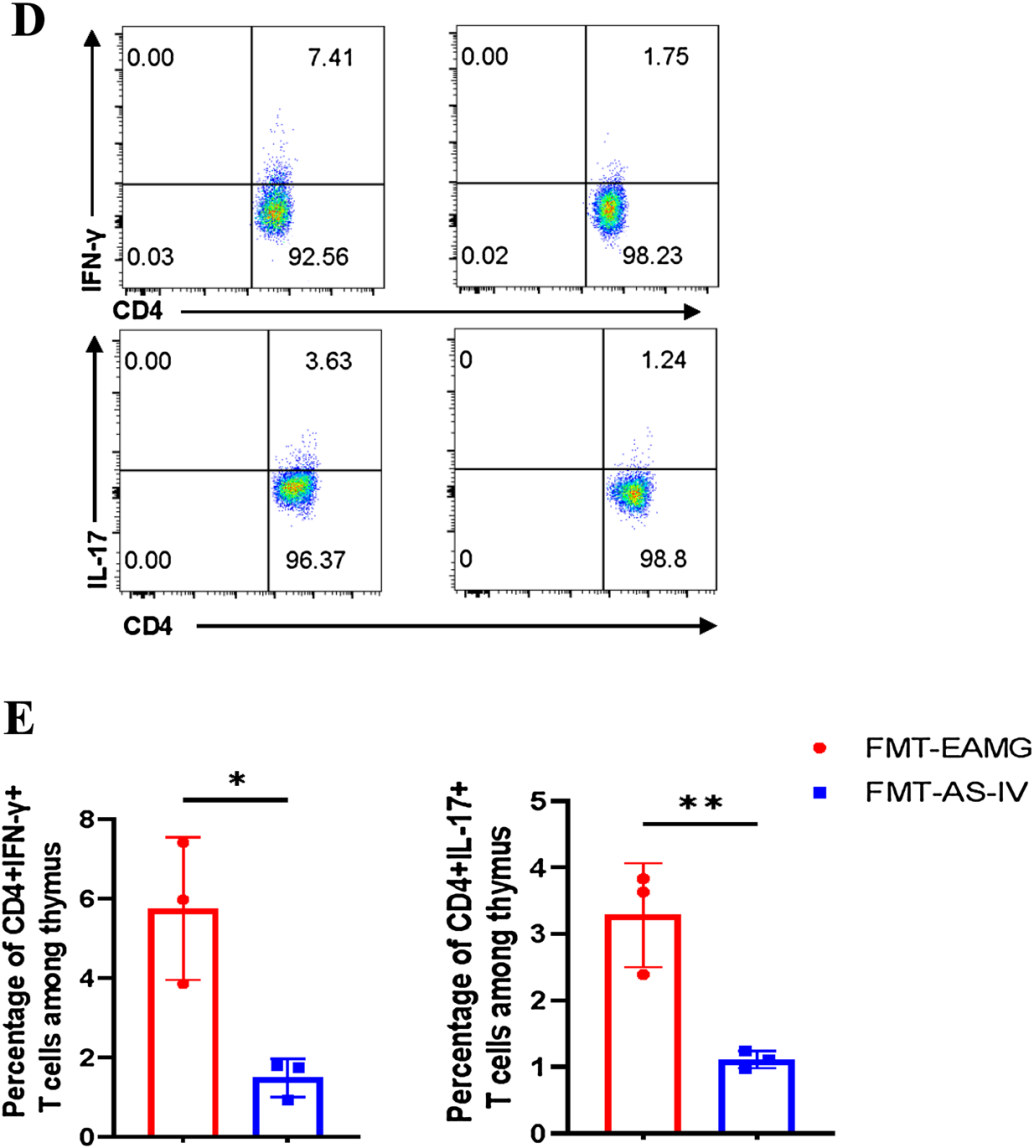
**A** After 4 weeks of FMT, the supernatant of mice was collected to determine the cytokines level of TGF-β, IFN-γ and IL-17. MNCs were collected and Th1 cells, Th17 cells of thymus and spleen and Treg cells of spleen were detected **(B**–**E)**. TGF-β, IFN-γ and IL-17 cytokines levels and the percentage of Treg, Th1, and Th17 cells in FMT EAMG group and FMT AS-IV group were compared (n = 3/group). Data are representative of two independent experiments. (*p < 0.05, **p < 0.01, ***p < 0.001, ****p < 0.0001) [5]

In addition, cytokine levels of IFN-γ and IL-17 in serum were significantly lower in FMT AS-IV group compared with FMT EAMG group (Fig. 10A). The alteration of gut microbiota of EAMG treated with AS-IV could down-regulate the Th1 and Th17 cell proportions in the spleen (Fig. 10B, C) and thymus (Fig. 10D, E) and up-regulated Treg cell proportions in spleen (Fig. 10B, C) in EAMG-induced mice. These results suggest that the alteration of gut microbiota by AS-IV affecting EAMG onset can modulate immunity and ameliorate EAMG severity. AS-IV might modulate the balance of Th1, Th17, Treg cells and cytokines in EAMG by altering the structure of intestinal flora, and consequently alleviate the severity of EAMG onset.

## Discussion

MG is an autoimmune disease usually associated with abnormal thymus involving thymic hyperplasia and thymoma. In abnormal thymus, the process of T cell maturation is impaired, resulting in an imbalance of T cell subsets. The imbalance of T cell subsets and changes in secreted cytokines activate the differentiation of B cells into effector B cells and plasma cells, leading to the production of pathogenic antibodies such as anti-AChR antibody [22, 23]. Previous studies have shown that MG is closely correlated with CD4 + T cells. Extensive data indicate that CD4 + T cells (Th1, Th17, and Treg cells) are involved in the development of MG and EAMG through a complex network of interactions among these cells and their cytokines [5]. Th1 cells produce pro-inflammatory cytokines, IFN-γ and IL-2, which activate antigen presenting cells for immune response amplification and mediate cytotoxic reactions. Th1 cells also induce differentiation of B cells that secrete immunoglobulin isotypes and IgG subclasses that bind and activate complement. Due to their roles in promoting inflammation and complement fixation, Th1 cells are good contributors to T cell–mediated and antibody-mediated autoimmunity [24]. Th1 cell-generated cytokines can play a key role in MG and EAMG pathogenesis, as they stimulate the production of pathogenic antibodies that exacerbate MG symptoms. Consequently, Th1 cells are necessary for inducing pathogenic anti-AChR antibody synthesis in EAMG [25, 26].

Th17 cells, which mainly secrete the pro-inflammatory cytokine IL-17, are categorized as a unique subset of CD4 + T cells. They play a significant role in the pathogenesis of experimental autoimmune diseases through promoting inflammatory responses, enhancing B cell function and releasing proinflammatory cytokines. In the context of MG pathogenesis, Th17 cells and IL-17 secretion have been found to be inextricably linked. For example, a study emphasized that IL-17 independent pathways contributed to the autoimmune response related to EAMG development [27]. Furthermore, a study have demonstrated a positive correlation between serum IL-17 levels, the degree of quantitative MG score, and anti-AChR antibody titers, indicating a more severe disease course [28]. Emerging evidence suggests that Th17 cells may play an important pathogenic role in the development of autoimmune pathology, which occurs when the function of Th1 cells is compromised. This phenomenon involves the mediation of T cells and complement-fixing antibodies [25]. Thus, Th17 cells and their associated cytokines are implicated in inducing anti-AChR antibody-mediated autoimmunity at the neuromuscular junction [29].

Treg cells are a distinct subpopulation of T cells with suppressive functions that play crucial roles in maintaining immune homeostasis and preventing autoimmunity. Treg cells, which suppress the immune response by increasing anti-inflammatory cytokines such as TGF-β and inhibit the function of other activated T cells [30], have been suggested as clinical indicators of MG [9]. Studies have shown that the frequency and function of Treg cells are impaired in MG patients, which was accompanied by reduced expression of Foxp3 and contributes to the pathogenesis of the disease [31–33]. In MG patients, especially those with thymus-related MG and without immunosuppressive therapy, the level and proportion of Treg cells were lower than those in healthy individuals [34]. The inhibitory effect of Treg cells on B cells can be attributed to their ability to regulate the cytokine environment and the function of antigen-presenting cells, primarily dendritic cells. This results in the inhibition of B cells differentiating into antibody-producing plasma cells, consequently reducing the production of anti-AChR antibodies.

Taken together, the severity and anti-AChR antibody titer in MG are closely related to the imbalance of Th1, Th17, and Treg cells.

AS-IV, a primary active ingredient of AM, has been shown to significantly affect CD4 + T cells such as Th1, Th17, and Treg cells and alleviate inflammation. These effects have been demonstrated in EAE, asthma and so on [17, 18]. Therefore, we hypothesized that AS-IV might improve MG by affecting CD4 + T cells. In this research, we identified the common target genes of MG disease and AS-IV compound by network pharmacology and KEGG enrichment analysis revealed the involvement of T cell receptor pathway and Th17 pathway among others. This finding indicated that AS-IV could affect MG via T cell-related pathways. This, to some extent, verified our conjecture that AS-IV improves EAMG by regulating CD4 + T cells.

Antibody titer tends to be associated with disease severity. By comparing CFA, EAMG, and EAMG + AS-IV group, we found that EAMG mice showed significantly increased serum anti-R97-116 IgG antibody excretion and worse muscle weakness symptoms than normal mice (CFA), including decreased grip strength, increased clinical muscle weakness score, weight loss. However, the anti-R97-116 IgG antibody titer and the conditions were significantly alleviated by AS-IV intervention. Further, flow cytometry and ELISA were used to analyze the levels of CD4 + T cells in spleen and thymus as well as the levels of IFN-γ, IL-17, and TGF-β in the serum of mice. Th1 and Th17 cells and their secreted cytokines (IFN-γ and IL-17) in EAMG were significantly increased, while Treg cells and their secreted cytokine TGF-β were significantly decreased, and AS-IV intervention could significantly change this trend. In addition, the alterations of the structure of gut microbiota in the three groups were compared after the test. Therefore, it can be inferred from our findings that AS-IV exerts its alleviative effects on EAMG by downregulating Th1 and Th17 cells, upregulating Treg cells, and reducing the titer of anti-R97-116 IgG antibodies.

In addition, in recent years, gut microbiota has been suggested as a possible pathogenesis of MG, indirectly influencing the symptoms and severity of MG patients by affecting host immunity. A study demonstrated that gut microbiota are the regulators of Th17/Treg balance in MG patients [29]. In this study, we investigated the impact of AS-IV on the gut microbiota of EAMG mice and explored whether AS-IV could improve EAMG by affecting the expression of CD4 + T cells and altering gut microbiota.

Diversity index is a measure of species diversity, in which alpha diversity index focuses on the number of species in locally homogeneous habitats, also known as the diversity within the biotic territory. Beta diversity index refers to the species composition of different habitat communities along the environmental gradient or the rate of species replacement along the environmental gradient, also known as inter-habitat diversity. Zheng Peng et al*.* found that the alpha diversity index of gut microbiome in MG patients was lower than that in healthy people, and the lower the alpha diversity index, the higher the severity of MG disease it is [35]. However, German Moris et al*.* showed the results with some differences. Their study showed that beta diversity of gut microbiome was different between MG patients with healthy people, but there was no difference in alpha diversity [36]. In this research, according to our results, there was no significant difference in the alpha diversity of bacterial communities among the three groups, while there was a significant difference in the beta diversity which means that there were significant differences in the principal component performance of gut microbiome among them.

At the level of phyla, Bacteroidetes and Firmicutes accounted for most of gut microbiota, and were the dominant phyla in gut microbiota. F/B ratio can be described as a kind of proinflammatory environment, and is thought to be associated with autoimmune diseases. In the intestinal tract, inflammatory bacteria destroy intestinal epithelial cells (IECs) and trigger an immune response, leading to immune disorders in autoimmune diseases. Multiple studies have shown that people with inflammatory bowel diseases, such as Crohn’s disease, have a reduced F/B ratio of gut microbiota compared to healthy people [26, 27]. MG patients had a lower F/B ratio than healthy people [28]. Our results were consistent (with significant differences) with a lower F/B radio in EAMG mice than in CFA mice, and AS-IV intervention increased the F/B ratio in EAMG mice. This suggests that AS-IV can raise the F/B ratio of gut microbiota. Thereby, AS-IV may ameliorate immune dysregulation in EAMG mice by modulating the abundance of Firmicutes and Bacteroidetes.

Clostridia, which are well-known producers of SCFAs and colonize the mucus layer near the epithelium, play an essential role in IECs [37]. Studies have shown that Clostridia increase the expression of 2, 3-dioxygenase and TGF-β1. This may promote the differentiation of primordial CD4 + T cells into antigen-specific colonic Foxp3 + CD4 + Treg cells, thereby increasing the frequency of Foxp3 + CD4 + Treg cells [38, 39]. A study found that the abundance of Clostridia in MG patients was significantly lower than that in healthy individuals [15]. In the current study, we obtained similar results that the abundance of Clostridia in the gut microbiota of EAMG was significantly lower than that of CFA, whereas the Clostridia recovered in the EAMG mice with AS-IV treated. Therefore, AS-IV increased the level of Treg cells in EAMG perhaps due to restoring Clostridia, thereby inhibiting the production of B cells and anti-AChR antibodies, and reducing the severity of EAMG. Meanwhile, FMT experiments demonstrated that Treg cells of FMT AS-IV mice increased significantly compared with FMT EAMG mice. This suggests that part of the mechanism of AS-IV altering EAMG is that it may affect the expression of Treg by changing the abundance of Clostridia, thus alleviating EAMG.

LEfSe analysis revealed that the richness of Bacteroides was greatly increased in the EAMG group compared with the CFA and EAMG + AS-IV groups. Some kind of protein of Bacteroides may act as a mimetic protein, generating antibodies that cross-react with self-epitopes, which may contribute to autoimmune conditions [40, 41]. However, the specific mechanism needs to be further studied. Oscillospira is negatively correlated with chronic inflammation. Previous studies have found that lower Oscillospira indicates higher severity in ulcerative colitis patients [42, 43]. Oscillospira in EAMG + AS-IV group was significantly higher than that in EAMG, and therefore, it may be that AS-IV reduces inflammation in EAMG mice. Besides, a study showed that treatment of EAMG with two lactobacillus and two bifidobacterium probiotic strains increased Treg cell levels and reduced disease severity [44]. In the current study, Lactobacillus was significantly reduced in EAMG, and Dong XQ et al*.* found that the abundance of Lactobacillus in MG was lower than that in healthy people [15], which was consistent with our study. However, AS-IV did not restore its abundance in this study.

FMT is a useful translational experimental model for assessing whether changes in the gut microbiome may be involved in the development of various diseases. In this study, we first proved that AS-IV can relieve the clinical symptoms of EAMG and change the imbalance of CD4 + T cells in vivo. However, whether AS-IV improves EAMG by regulating the structure of gut microbiota or not has not been confirmed. Therefore, we conducted FMT tests. The bacteria in EAMG + AS-IV group were intragastrically administered to EAMG Abx-treated mice, and it exhibited a reduction in clinical score, a decrease in anti-R97-116 IgG titers, as well as a decrease in the proportions of Th1 and Th17 cells and an increase in the proportion of Treg cells compared with FMT EAMG group. This proved that changes in the gut microbiota of EAMG interfered by AS-IV can improve EAMG and regulate CD4 + T cells of EAMG.

In the future, we will conduct a controlled study on different concentrations of AS-IV to explore the alteration of effects in improving EAMG, so as to pave the way for the research of AS-IV in the treatment of EAMG.

## Conclusions

Taken together, through network pharmacology, we found that the intervention of AS-IV on MG may be related to T cell pathways. AS-IV alleviates EAMG by regulating Th1, Th17 and Treg cells, and this effect was probably due to the alteration of gut microbiota in EAMG. Therefore, AS-IV may be effective in the treatment of MG, and its further mechanism and specific pathway still need to be further studied.

## Data Availability

The research data used to support the findings are available from the corresponding author upon request.

## Abbreviations

AS-IV: Astragaloside IV
MG: Myasthenia gravis
EAMG: Experimental autoimmune myasthenia gravis
AM: Astragalus membranaceus
FMT: Fecal microbial transplantation
F/B: Firmicutes/Bacteroidetes
Th: T helper cell
Treg: Regulatory T cell
SCFA: Short-chain fatty acid
MNC: Mononuclear cell
KEGG: Kyoto Encyclopedia of Genes and Genomes
CFA: Complete Freund’s adjuvant
IFA: Incomplete Freund’s adjuvant
LEfSe: Linear discriminant analysis effect size
IECs: Intestinal epithelial cells
Foxp3: Forkhead box protein 3
OTUs: Operational taxonomic units
